# The design of a purpose-built exergame for fall prediction and prevention for older people

**DOI:** 10.1186/s11556-015-0157-4

**Published:** 2015-12-08

**Authors:** Hannah R. Marston, Ashley Woodbury, Yves J. Gschwind, Michael Kroll, Denis Fink, Sabine Eichberg, Karl Kreiner, Andreas Ejupi, Janneke Annegarn, Helios de Rosario, Arno Wienholtz, Rainer Wieching, Kim Delbaere

**Affiliations:** Institute of Movement and Sport Gerontology, German Sport University Cologne, Am Sportpark Muengersdorf 6, Cologne, 50933 Germany; Neuroscience Research Australia, University of New South Wales, Barker Street, Randwick, Sydney, New South Wales 2031 Australia; Assistive Healthcare Information Technology Group, Austrian Institute of Technology, Donau-City-Strasse 1, Vienna, 1220 Austria; Personal Health Department, Philips Research Europe, High Tech Campus 34, Eindhoven, 5656AE The Netherlands; Biomedical Research Networking Center in Bioengineering, Biomaterials and Nanomedicine (CIBER-BBN), Healthcare Technology Group, Valencia, Spain; Institute of Biomechanics of Valencia, University Polytechnic of Valencia, Edificio 9C Camino de Vera s/n, Valencia, 46022 Spain; Kaasa Solution GmbH, Flinger Str. 11, Düsseldorf, 40213 Germany; Institute for Information Systems, University of Siegen, Hölderlinstrasse 3, Siegen, 57076 Germany

**Keywords:** Exercise, Strength, Balance, Older people, Technology, Exergame, Fall risk, Fall prevention

## Abstract

**Background:**

Falls in older people represent a major age-related health challenge facing our society. Novel methods for delivery of falls prevention programs are required to increase effectiveness and adherence to these programs while containing costs. The primary aim of the Information and Communications Technology-based System to Predict and Prevent Falls (iStoppFalls) project was to develop innovative home-based technologies for continuous monitoring and exercise-based prevention of falls in community-dwelling older people. The aim of this paper is to describe the components of the iStoppFalls system.

**Methods:**

The system comprised of 1) a TV, 2) a PC, 3) the Microsoft Kinect, 4) a wearable sensor and 5) an assessment and training software as the main components.

**Results:**

The iStoppFalls system implements existing technologies to deliver a tailored home-based exercise and education program aimed at reducing fall risk in older people. A risk assessment tool was designed to identify fall risk factors. The content and progression rules of the iStoppFalls exergames were developed from evidence-based fall prevention interventions targeting muscle strength and balance in older people.

**Conclusions:**

The iStoppFalls fall prevention program, used in conjunction with the multifactorial fall risk assessment tool, aims to provide a comprehensive and individualised, yet novel fall risk assessment and prevention program that is feasible for widespread use to prevent falls and fall-related injuries. This work provides a new approach to engage older people in home-based exercise programs to complement or provide a potentially motivational alternative to traditional exercise to reduce the risk of falling.

## Background

The prevention of falls and mobility-related disability among older people is an urgent public health challenge worldwide [1]. The risk of falling is linked to several factors which include gait instability, muscle weakness, balance impairment, slow reactions, visual deficit, the type and number of medications, cardiovascular conditions, cognitive impairment, and the history of falls [2, 3]. Evidence from systematic reviews and meta-analyses suggests that tailored, multifactorial programs with an overall focus on exercise are more likely to prevent falls [1]. However, in order to promote uptake and adherence, identification of enjoyable and engaging falls prevention programs are needed [4].

Serious games are digital games used for learning or training purposes and have become a popular area for research [5, 6]. These types of games can provide an opportunity to deliver health messages, and to motivate and guide people to make lifestyle changes [5–7]. The utilization of digital game technology has increasingly been used to deliver exercise programs and to facilitate health rehabilitation [5, 6]. Commercially-available devices such as the Nintendo™ Wii (2005) and Microsoft Xbox Kinect® (2010) can monitor movement via inexpensive technologies within a home-based setting. Using technology to deliver exercise training may enable greater choice in preferred exercise options, increase convenience and accessibility, and enhance a greater level of engagement [8]. Further, the use of technology allows for immediate performance feedback, extended monitoring and analysis, as well as optional interaction with clinicians and/or peers, which allows people to obtain assistance when needed. Therefore, there is potential for technology-based solutions to reduce costs while maintaining individualised high-quality healthcare.

The main objective of the Information and Communication Technology-based System to Predict and Prevent Falls (iStoppFalls) project was to develop and evaluate innovative home-based technologies for fall risk assessment, continuous monitoring and prevention of falls in community-dwelling adults. This approach will enable individualised exercise and education programs coached by the iStoppFalls system, using unobtrusive technology. This paper aims to present and discuss the development of the iStoppFalls system towards the prediction and prevention of falls by monitoring mobility-related activities and risk factors for falls in everyday-life and delivering a home-based exercise program.

## Methods

**Fig. 1:**
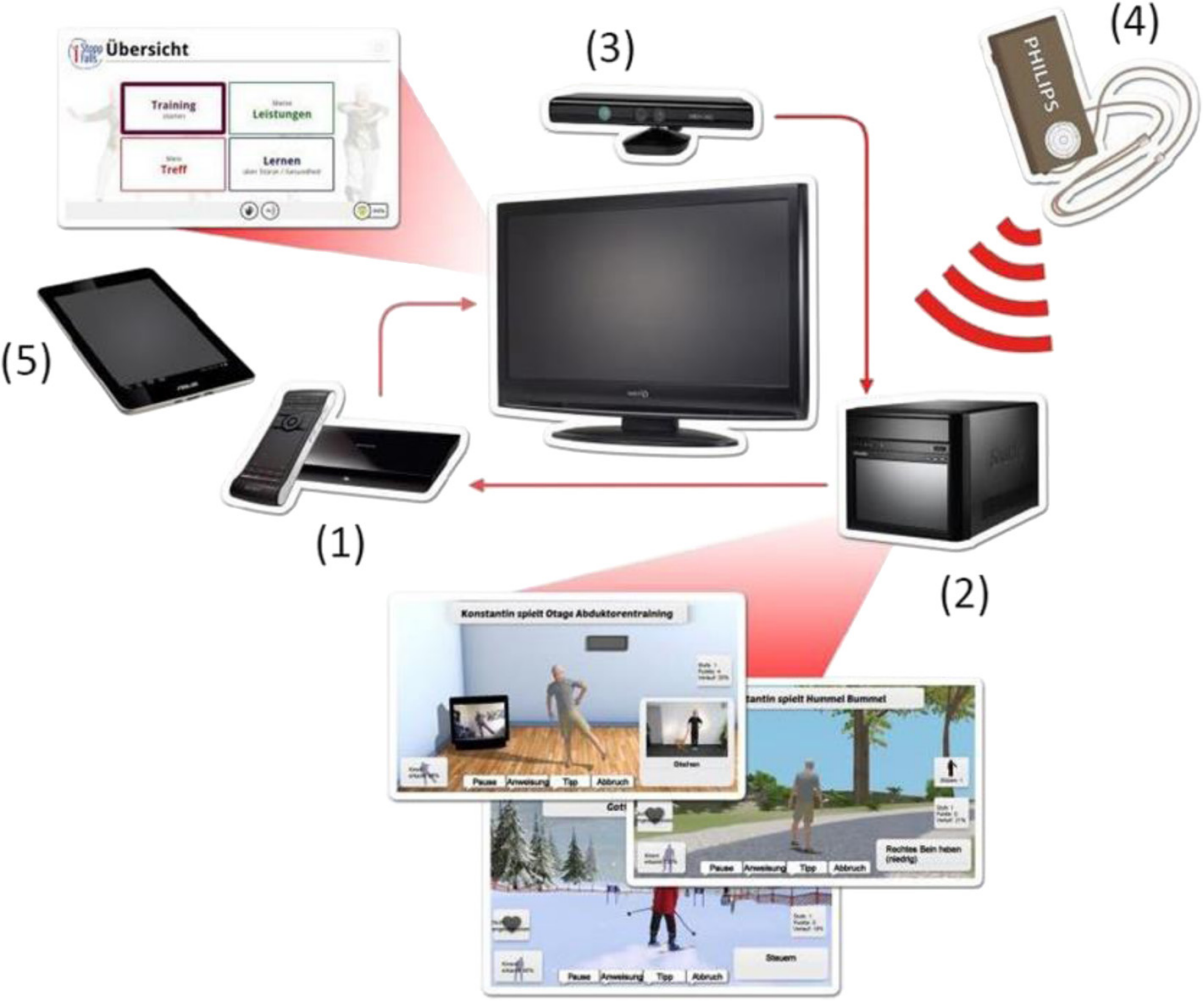
Overview of the technologies used in iStoppFalls: (**1**) set top box (iTV), (**2**) mini-PC (exergame), (**3**) Kinect (gesture/voice), (**4**) Senior Mobility Monitor, (**5**) tablet (diary, control)

**Fig. 2:**
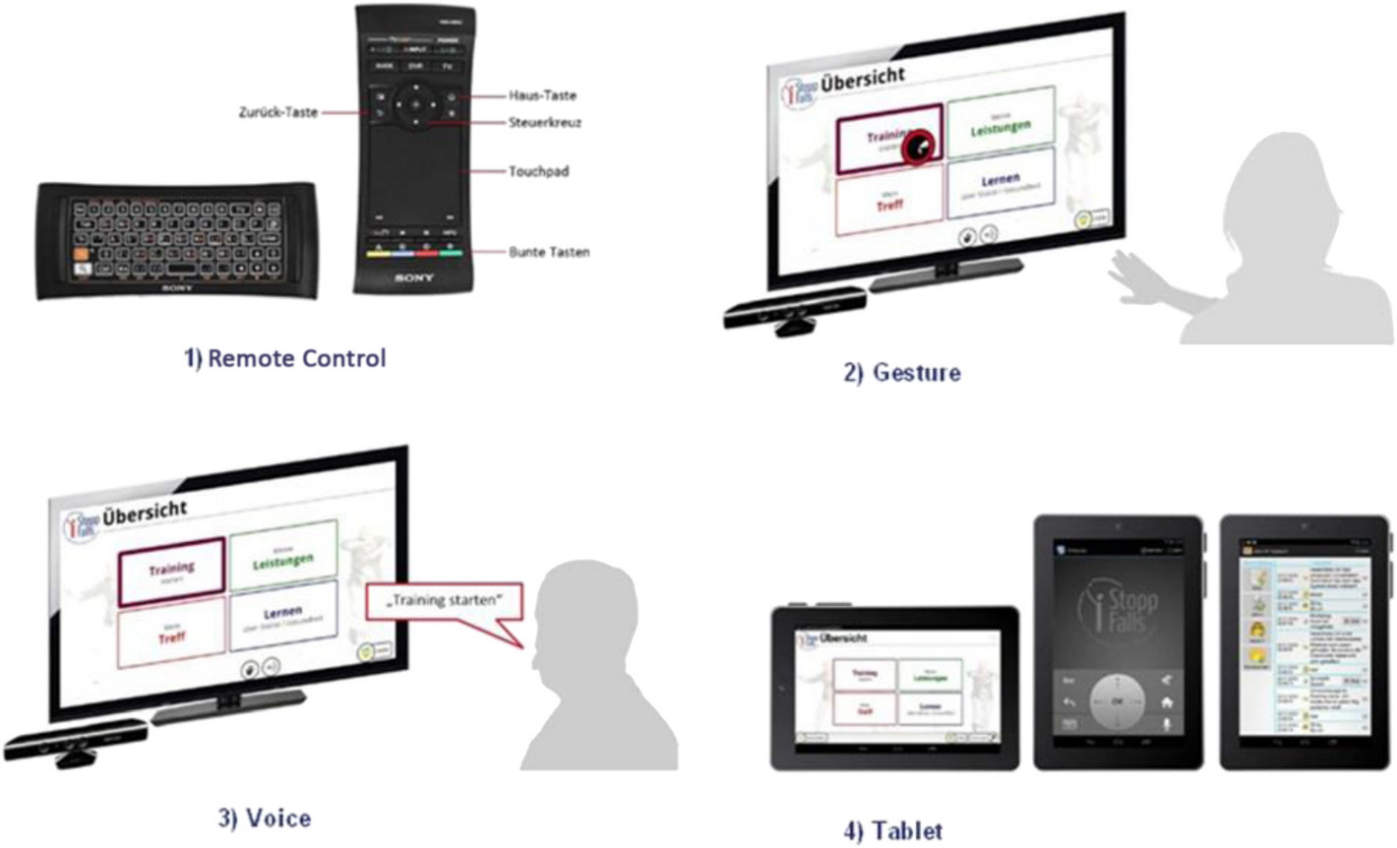
iStoppFalls interaction components through the iTV system. The user communicates with the iStoppFalls program by (**1**) remote control, (**2**) gesture, (**3**) voice or (**4**) tablet

**Fig. 3:**
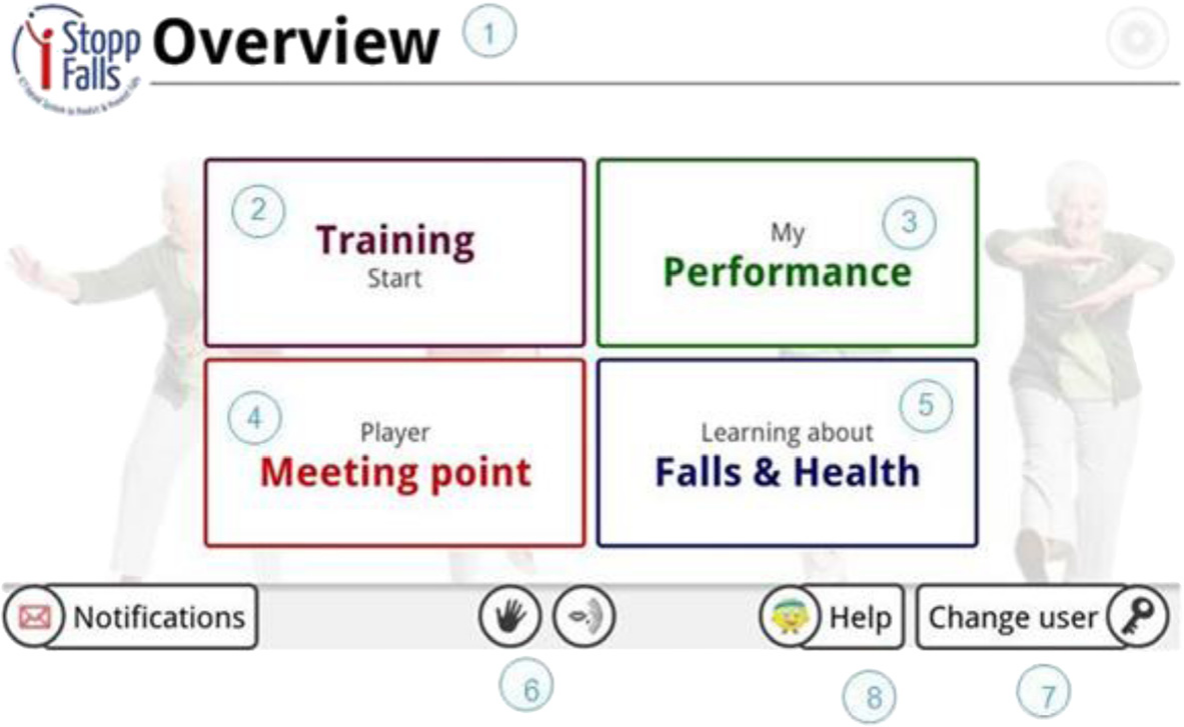
The iStoppFalls menu is the main page for all users. Each number is associated to the image and provides an overview of the function (**1**). Page header. (**2**). Training: The area of training. The user can exercise or determine their risk of falling. (**3**). Performance: The user can view their feedback and results. (**4**). Meeting Point: The user can communicate with other users who use the system. (**5**). Falls & Health: The area of learning, educational material and information on fall risks in everyday life, and how to reduce this risk. (**6**). Gesture and Voice Recognition: Two buttons to activate the gesture and/or voice control. (**7**). Change User: The user can either log out of the program or start with a different user account (**8**). Help: The user can find help in this section for the most common problems and how to use the system

The emphasis of the iStoppFalls project is not on laboratory research but on active implementation of existing technologies and evidence-based fall prevention strategies for older people living in the community. The iStoppFalls system uses innovative technologies that can be integrated in people’s homes at a low cost (Fig. 1). The Philips Senior Mobility Monitor (SMM) is a research prototype that monitors activity and mobility in daily life regularly and unobtrusively. The Microsoft Kinect and PC deliver home-based exercise through a newly developed fall preventive exercise game (exergame) on the home television. A Knowledge Based System (KBS) integrates information from the SMM and Kinect/PC system to provide feedback on performance and progression, and enables secure data management together with a web-based interface for researchers. The Google TV set top box by Sony was used to integrate the SMM, Kinect/PC system and KBS and delivers the iStoppFalls program through an innovative interactive television (iTV) system. The iTV system enables access and facilitates communication between the user and the iStoppFalls program. Through the iTV system, the user communicates with the iStoppFalls program by voice, gesture, remote control or tablet (Fig. 2). The iStoppFalls program is accessed through the iStoppFalls menu (Fig. 3) which comprises of four main components: *Training*/*Physical tests* (balance exergames, strength exercises and fall risk assessment), *Performance*/*Feedback* (balance exergame results, strength exercise results, falls risk assessment results and SMM activity profile), *Learning*/*Education* (education fact sheets on falls risk factors) and *Meeting point* (private social network platform for iStoppFalls users) (Fig. 3). A training diary and study questionnaires can also be accessed via the tablet computer.

Following a scoping literature review and expert consultations, the content of the iStoppFalls program was designed to predict and prevent falls using technological solutions: (i) fall risk assessment to identify common fall risk factors; (ii) exercise program to improve balance and muscle strength; (iii) feedback on performance, progression, general activity and change in falls risk factors; (iv) education on modifiable and non-modifiable falls risk factors; (v) social media platform to provide users with the option to communicate with and find support from other users.

## Results

### Fall risk assessment

The iStoppFalls Fall Risk Assessment (FRA) tool has been developed based on existing evidence-based models for falls prediction, such as QuickScreen^©^ [9]. The QuickScreen^©^ assessment tool is an externally validated, reliable, and feasible falls risk assessment that can predict multiple falls with an accuracy of 72%, and assist with guiding interventions in community-living older people [9]. The iStoppFalls FRA also includes components of other validated sensorimotor assessments for falls prediction in community-living older people such as the Short Physical Performance Battery (SPPB) [10], the Iconographical Falls Efficacy Scale (Icon-FES) [11], and the Choice Arm and Stepping Reaction Time (CSRT) test [12]. The iStoppFalls FRA consists of questions relating to previous falls, medication, vision, peripheral sensation, a concern about falling (COF) questionnaire and four physical assessments including static balance tests, sit-to-stand (STS) test, as well as arm and stepping reaction time tests (Table 1). Scores on the assessments assisted to monitor and track performance of the user over time.

**Table 1:** iStoppFalls fall risk assessment

Type	Section	Origin	Element	Result	Cut-off
Question	Previous Falls	QuickScreen [9]	Have you had one or more falls in the previous 12 months?	Yes/No	Yes
Question	Medication	QuickScreen [9]	How many medications do you take each day? Do you currently take any medications to help you sleep?	Number Yes/No	>4 Yes
Question	Vision	QuickScreen [9]	Do you wear multi-focal glasses outside?	Yes/No	Yes
Question	Peripheral Sensation	QuickScreen [9]	Do you have painful feet?	Yes/No	Yes
Question	Concern about Falling	Icon-FES [11]	10 questions using pictures to illustrate the daily activity	1–4 Likert scale	>13
Physical	Reaction time	New	Choice arm reaction time test	Pass/Fail	>800 ms
Physical	Reaction time	CSRT [12]	Choice stepping reaction time test	Pass/Fail	> 800 ms
Physical	Balance	SPBB [10]	Semi-tandem test	Pass/Fail	<30 s
Physical	Strength	QuickScreen/SPPB [9, 10]	Sit-to-stand test	Pass/Fail	>12 s

#### iStoppFalls FRA questionnaire

Having a history of falls is a risk factor for falling [2]. Therefore a fall history in the previous 12 months was assessed. Further, several studies have demonstrated a relation between drug use and falls among older people. Hence, medication use was included in the questionnaire; such as taking four or more medications per day and taking psychotropic drugs [1, 9]. Questions regarding previous falls and medications were taken from QuickScreen^©^ [9].

In contrast to the QuickScreen^©^ assessment, vision and foot problems were assessed in the iStoppFalls FRA questionnaire. Decreased contrast sensitivity and depth perception, especially in combination with wearing multifocal eyeglasses, is important for maintaining balance during daily activities and detecting and avoiding environmental hazards [13]. Reduced peripheral sensation, inappropriate footwear, foot conditions and feet pain have shown to impair balance and increase the risk of falls [3, 13]. The iStoppFalls FRA also included a measure on concern about falling. Previous research has demonstrated that concern about falling is strongly related to future falls [14]. Concern of falling was assessed with the 10-item Iconographical Falls Efficacy Scale (Icon-FES), which uses pictures of 10 daily activities associated with falls on a four-level Likert scale [11]. Higher scores indicate higher levels of concern.

#### iStoppFalls FRA physical assessments using sensor-based technology

The iStoppFalls FRA physical assessments were selected from a range of validated assessments including QuickScreen^©^ [9], SPBB [10] and CSRT. The four physical assessments address a range of domains shown to significantly increase fall risk, i.e. balance, functional mobility and reaction time [15, 16]. The balance test assesses an individual’s ability to stand in three separate foot positions: semi-tandem, near-tandem and full-tandem stance for 30 s. Each test provides a measure of lateral stability which is crucial for maintaining balance and preventing sideways falls. Previous studies have reported that poor performance in this test is associated with an increased risk of falls in older people [17, 18]. The STS test with five repetitions is a functional mobility measure of composite lower limb strength, speed and balance. Previous studies have reported that the STS test is a significant predictor of falls in older community-living people. The arm and stepping reaction time tests measure the ability to react and ‘hit’ or ‘step’ to a visual stimulus. Previous studies have shown that a slowed reaction time is a significant predictor of falls [13]. The stepping reaction time test also gives an indication of reduced stepping ability which is a known risk factor for falls as a reduced ability to manoeuvre the feet in response to a balance-disturbing stimulus increases the likelihood of a fall [15].

#### iStoppFalls FRA as a fall risk screening tool

**Fig. 4:**
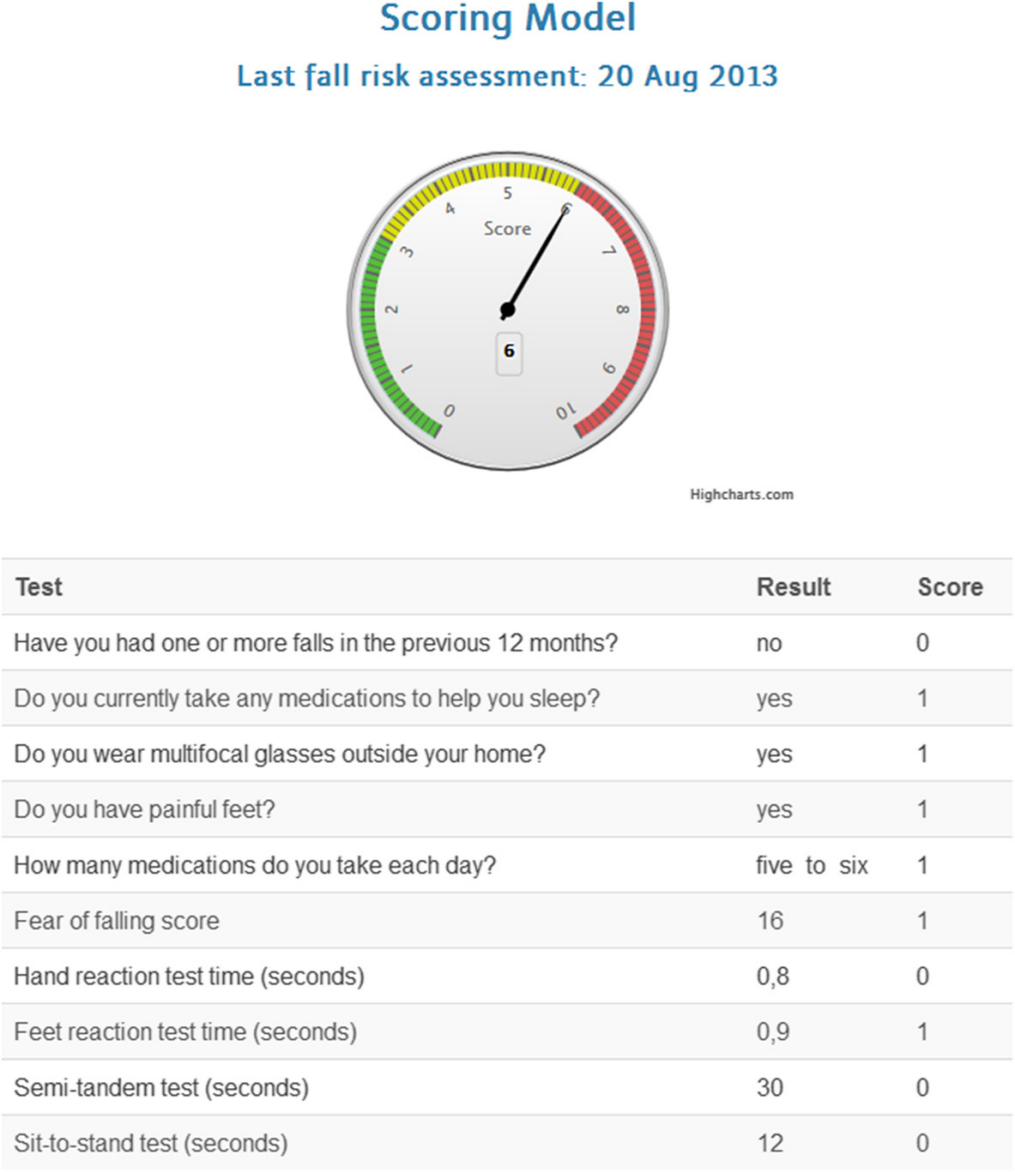
Fall risk feedback based on 10 items

The result from each assessment is adapted to have a binary decision outcome. Most questions are phrased to give a ‘Yes’/’No’ outcome and each physical assessment has a performance cut off to give a ‘Pass’/’Fail’ outcome. If a user indicates ‘Yes’ on the questions or their performance is poorer than a particular cut off (Table 1), the iStoppFalls FRA determines that the individual demonstrates that risk factor. A score of 1 is given for each risk factor the individual demonstrates. Following completion of the FRA, the iStoppFalls system displays a total fall risk factor score with a breakdown of results and explanations for each assessment. As there are 10 assessments and therefore 10 risk factors, an individual’s fall risk score can range from 0 to 10 (Fig. 4). A result value of 0 indicates the user is at a lower risk of falls. A fall risk score of 10 indicates the individual is at a higher risk of falling as they have demonstrated all of the assessed risk factors.

### iStoppFalls exercise program

Exercise targeted to improve balance, muscle strength and reaction time has proven to enhance mobility and prevent falls in community-dwelling older people [19]. A literature search was conducted to identify suitable evidence-based fall prevention exercise programs for the iStoppFalls system. The iStoppFalls exergame program was modelled on the Otago Exercise program (OEP) for general strength and balance training [20] and the Weight-Bearing Exercise for Better Balance (WEBB) intervention for functional balance and mobility training [21]. The OEP is an evidence-based cost-effective home-based exercise program using principles of self-management to reduce falls and fall-related injuries [20]. This program has been shown to improve participants‘ strength and balance, as well as confidence in carrying out everyday activities without falling [22]. The WEBB is a challenging, safe, evidence-based physiotherapy program for older people [21]. Both programs were analysed for exercise and training principles which could be incorporated into the iStoppFalls exercise program and adapted to be delivered via the iStoppFalls system. The availability of motion capture technology allowed the inclusion of exergames to train balance (combined with dual-task training once higher levels were reached) as well as traditional strength exercises. The iStoppFalls exercise program consists of strength exercises to improve lower limb strength and three exergames to improve balance and dual-tasking in a virtual environment. Further, the novel design of the iStoppFalls exergames aims to increase uptake and compliance of training, because many older people, especially frailer ones, are reluctant or unable to attend exercise sessions outside of their home. Participants were encouraged to undertake a minimum of three balance sessions of 40 min and three muscle strength sessions of 20 min per week. Furthermore, the study protocol suggested a 10 min warm-up of balance exercises prior to undertaking strength training sessions. Weekly training sessions should total about 120 min for balance training and 60 min for strength training. This constitutes approximately 50 h of strength and balance training over a period of 16 weeks which follows the recommended exercise dose for falls prevention [19].

#### Strength exercise program

**Fig. 5:**
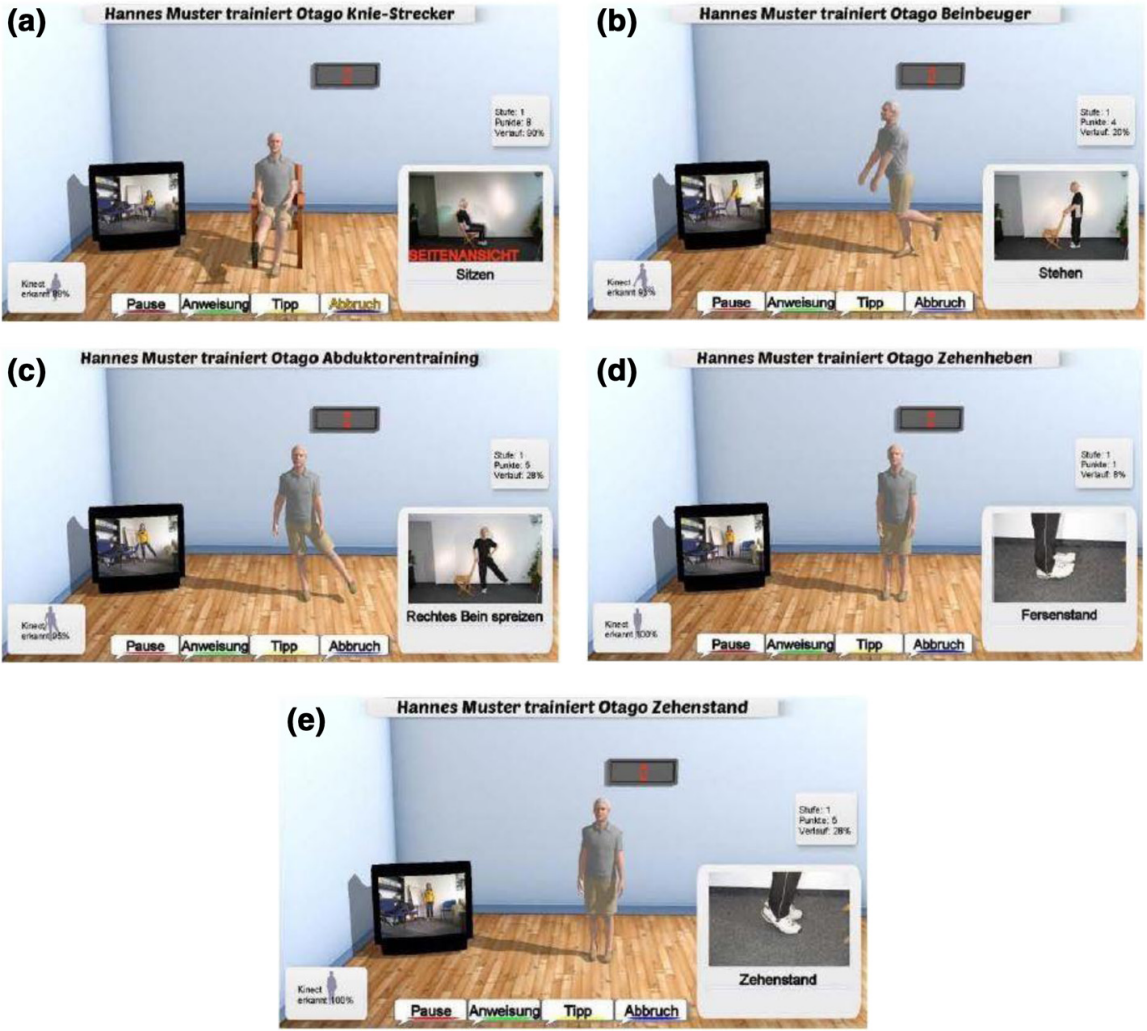
Otago Exercise Program: The images depict the avatar/user executing the Otago exercises (**a**) knee extension, (**b**) knee flexion, (**c**) leg abduction, (**d**) toe raises, (**e**) calf raises. There is a demonstration via the icon on the bottom right hand side of the screen. On the right side of the screen, the users are able to see themselves on the television screen. The four buttons at the bottom of the screen (pause, instructions, tips, and abort) can be selected by the users to execute the command

The strength exercises focus on major lower limb muscles which are important during functional movements, walking, and recovering balance including seated knee extension (Fig. 5a), standing knee flexion (Fig. 5b), standing hip abduction (Fig. 5c), toe raises (Fig. 5d) and calf raises (Fig. 5e). To ensure safety, the iStoppFalls program recommends holding on to at least one chair for support. As a general guide from the OEP, the initial intensity of strength exercises should aim to be prescribed at a moderate intensity [23]. However, as delivering strength exercise through home-based technology to older people is innovative, all exercises begin at the lowest intensity to enable the user to familiarise themselves with the technology, the real-time feedback and the quality of their movements. Each exercise gradually increases in difficulty as the program progresses in order to maintain at least a moderate intensity. The strength exercises are progressed through increasing the level of difficulty, increasing number of repetitions, increasing number of sets (maximum 3 sets per exercise) and/or adding ankle cuff weights (1 kg, 2 kg or 3 kg) to the exercise. The iStoppFalls KBS ensures that the user is able to complete two sets of 10 repetitions of each exercise before progressing to a more challenging intensity. The progression of each exercise uses no more than one of the above mentioned methods.

#### Balance exergames

The iStoppFalls balance exergames target three key elements identified from the OEP and WEBB programs: i) weight shifting, ii) knee bending and iii) stepping. From these aspects, three games were created: *Hills n*‘ *Skills*, *Balance Bistro* and *Bumble Bee Park*. The three exergames aim to improve dynamic balance and stability by practicing activities relevant to functional tasks. To effectively improve balance and reduce falls, the exergames need to be moderate to highly challenging for each individual [19]. Progression of each game includes reducing upper limb support, narrowing base of feet support, decreasing speed of movement (e.g. during weight shifting), including movement of arms (e.g. reaching), and combining the three key elements of weight shifting, knee bending and stepping. In addition to the balance training, each game contains a cognitive component by providing dual-tasking situations which comprise of mathematical equations, memorizing a coloured object or recalling the number of displayed objects. The difficulty of the cognitive component is also increased to add another level of challenge.

**Fig. 6:**
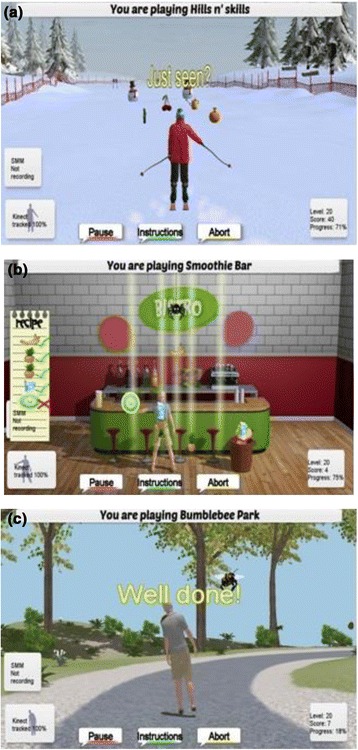
Exergames: (**a**) Hills n’ Skills, (**b**) The Bistro and (**c**) Bumble Bee Park

The *Hills n’ Skills* game is a virtual skiing game where the user must navigate their way down a ski slope (Fig. 6a). This exergame primarily targets knee bending, weight shifting and reaching to train functional balance ability. The user is required to shift their body weight and lean left or right, while manoeuvring between gates, collecting coins, avoiding obstacles (snowmen) and completing a memory task. The gates, coins and obstacles serve as motivational tools while also enabling a varied progression of the game. The memory task (Table 2) requires the user to perform mathematical equations and memorizing coloured objects. Responses are given by the user with reaching to one of three responses provided on the screen.

**Table 2:** Balance exercises and game components

iStoppFalls exergames	Task	Exercise	Game component
Bumble Bee Park	Stepping	Marching on the spot which moves the avatar/user around the footpath of the park.	To travel around the footpath from start to finish.
	Reaching	Reaching enables the avatar/user to select the correct answers from the memory tasks.	To select the correct answer based on the memory tasks.
	Leaning	Leaning to the left or right allows the user to avoid bees flying around.	Avoiding hazards (bumble bees).
	Cognition/dual task	Users reach upwards to select the correct answer.	Mathematical equations comprising of two digit sums and are either subtraction or addition. Colour objects are shown initially and then four choices are presented to the user. Then the correct object should be selected. Participants are instructed to count the number of items prior to starting the game. They are then shown four answers and then select the correct answer which relates to the number of items counted.
Hills ‘n’ Skills	Knee bending	Knee bending starts the game, and by bending the knees further, the avatar skis down the slope quicker.	To ski down the slope at varying speeds – depending on the depth of the knee bend.
	Leaning	Leaning to the left or right allows the user to avoid snowmen, or to pass gates.	Avoid snowmen, travel through gates and collect coins.
	Reaching	Reaching enables the avatar/user to select the correct answers from the memory tasks.	Select the correct answer based on the memory tasks.
	Cognition/dual task	Users reach upwards to select the correct answer.	Two memory tasks implemented.
			Mathematical equations comprising of two digit sums which are either subtraction or addition.
			Colour objects are shown initially and then four choices are presented to the user. The correct object was then selected.
Balance Bistro	Stepping	Side stepping across the screen to collect ingredients falling from the ceiling across three planes (left, centre, right).	Step across three planes (left, centre, right) on the Bistro floor.
	Reaching	Reaching enables the avatar/user to select the ingredients.	Collect as many ingredients as possible in the allocated time.
			Collect the specified ingredients in the correct order in the allocated time.
	Cognition/dual task	Users step to avoid objects	Avoid spiders and ingredients that are not part of the recipe.

The *Balance Bistro* is a food catching game where the user must ‘catch’ certain food items with a basket (Fig. 6b). This exergame requires people to step in different directions, shift their weight and reach to the left and right with their arms. The user must step left and right, shift its body weight appropriately while collecting food items (e.g. ham, cheese, bread and milk) that fall down from the ceiling of the virtual room. As the user progresses, the rate and number of falling food items increase and the objective changes from catching as many items as possible to following a recipe list to make a sandwich or smoothie. In addition cookies may fall down on the far left or right side of the screen which can be collected by stepping left or right, leaning and reaching out with an arm. An aspect of inhibition was added to the game through dropping spiders. The user must step and lean out of the way to avoid the spider which would freeze a column of falling food for a short period of time.

The *Bumble Bee Park* is an exergame where the user ‘walks’ around a footpath and avoids moving obstacles (i.e. bumble bees) by stepping and/or leaning (Fig. 6c). The user marches on the spot to make the avatar ‘walk’ on the screen. The faster the marching pace, the quicker the user progresses around the pathway. The difficulty of the exercise is progressed by increasing the step height which requires the user to lift their feet higher up off the floor. As the user ‘walks’ around the pathway, he/she encounters bumble bees which fly towards them. The user must lean their body to the opposite side to avoid colliding with the bee.

### Feedback

Providing feedback to users by monitoring and tracking their performance is an important element to maintain and increase the level of activity and motivation [8]. The iStoppFalls program provides a variety of feedback to the user such as fall risk assessment results, exercise scores and walking distance estimated based on the SMM data.

Following completion of the iStoppFalls FRA, the user is provided with feedback in three formats: a graph indicating the individual overall fall risk score, a written explanation of the results from each assessment and recommendations for maintaining or improving their performance. The overall score is visually represented to the user by a gauge which is colour-coded (green – low risk, yellow – medium risk, red – high risk) (Fig. 4). The feedback from the iStoppFalls FRA allows for the identification of people who are at a high risk of falling and the ability to determine which specific factors contribute to that risk.

Participants can gain additional feedback by accessing their training results through the ‘My Performance’ menu options on the iTV system. Following the completion of the balance exergames, users are provided with immediate feedback on the time it took to complete the game (seconds), the performance of the additional tasks (e.g., number of items collected, number of obstacles hit) and the total score they achieved. Feedback from each of the exergames can be seen through a series of charts, figures and tables displaying personal high scores, number of games played, and highest score achieved across all study participants. Additional features include achievement awards as a motivational tool to exceed their current activity levels. An achievement award is given when the user exceeds his/her personal best scores.

**Fig. 7:**
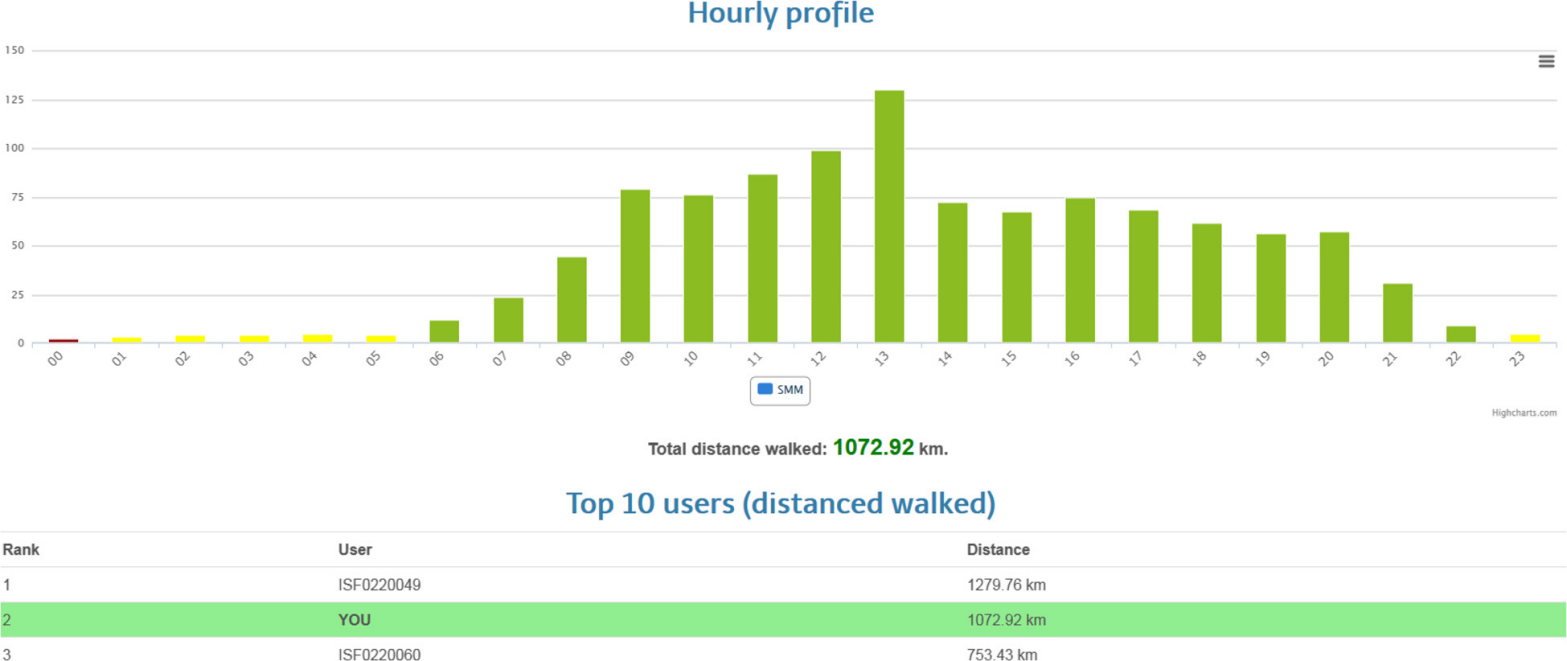
Senior Mobility Monitor activity profile: This visualization shows a daily activity profile of a user. Green bars mark high activity, yellow medium and red low activity. Moreover, a high score enables users to compare their daily activity to other users

Feedback from the SMM provides users with distance walked (km). This information is reported as a daily overview presented as a bar graph broken down into hourly segments. Displaying the data in this manner allows the user to visualise their total daily activity, to identify the time of day when the user was most active and to detect changes in the user’s activity patterns during the study period (improved, maintained or reduced activity levels). A chart is available for viewing each week reflecting the previous seven days of activity (steps and distance data). Figure 7 illustrates how data is presented to the user based on the distance walked every day. In addition to information on distance walked, the SMM provides the KBS with information on the number of chair rise transfers in daily life [24]. This measure of mobility provides additional insight into the fall risk of participants.

### Education material

Several studies have shown that falls prevention education in addition to a multifaceted intervention can reduce the risk and/or rate of falls [1]. As part of the iStoppFalls program, educational fact sheets offered information on factors that contribute to an increased risk of falling and on falls prevention strategies (i.e. general health, activity, lifestyle) via the iTV system and tablet computer. The falls prevention education topics are centred on known risk factors for falls, evidence-based fall prevention interventions, and, in areas where more research is required, further good practice principles. The content of the iStoppFalls educational material is largely based on the New South Wales Ministry of Health (Australia) ‘Staying active and on your feet’ booklet [25]. This information was then updated with recent research and evidence in active ageing and falls prevention. All documents included in the education component of the iStoppFalls program are listed in Table 3.

**Table 3:** Topics and domains of the educational material

Topic	Domain	Information
General Information	Understanding Falls	A general introduction on fall prevention and fall related injuries. Current statistics and research are summarised and known modifiable risk factors are highlighted.
Information on fall risk factors and fall prevention strategies	Exercise and Balance	Explains the benefits of exercise in the prevention of falls, states the recommended level of physical activity, includes tips to increase incidental activity and lists types of balance and aerobic exercise.
	Healthy Eating	Covers why diet, hydration, calcium and vitamin D are important. This topic is guided by good practice principles as there is limited evidence in this area.
	Your General Health	Information on how health can impact on balance and falls including acute and chronic health conditions, cardiovascular health, aspects of mental health, arthritis and incontinence.
	Vision	Outlines common changes in vision and how they can affect the ability to see, react and maintain balance (i.e., multifocal glasses or other eye conditions).
	Feet and Footwear	Describes how feet and common foot problems experienced by older people can impair balance. Includes why footwear is important for maintaining balance and outlines features of a ‘safe’ shoe.
	Bone Health and Fractures	Highlights why bone health and preventing fractures are important. This topic covers aspects of nutrition, exercise, prescription medications, and prevention of osteoporosis and hip protectors.
	Fear of Falling	Discusses why fear of falling can increase the risk of having a fall and how it can affect daily and physical activity levels, balance and confidence.
	Medicines	Describes why and how certain medications affect balance and increase risk of falling.
	Environmental Hazards	Information on potential hazards and risks both inside and outside the home are discussed. How and why certain surroundings can be a risk factor is outlined. Simple and more sophisticated home modifications are described.
	Emergency Plan	Describes why having an emergency plan is important and provides simple instructions to follow after a fall. Provides tips to assist them getting up or to get help after a fall.
Checklist	Health and Lifestyle	Questions relating to each of the education topics are asked to help individuals reflect on and identify their own fall risk factors.
	Home Safety	Designed to help older people follow basic home safety guidelines and identify aspects of their home that might increase their risk of a fall.
Quiz	Fall Quiz	Reflects on the most important points covered by the education fact sheets.

### Social media platform (SMP)

The SMP was designed, developed and integrated into the iStoppFalls system to provide the users with additional forms of communication and support between each other. The SMP allowed users to upload a profile (including picture) and post short messages which could be seen by other participants who uploaded a profile on the SMP. Additionally, the SMP allowed participants to post their exergame scores. It was anticipated that the SMP would increase adherence to the iStoppFalls system as participants are able to socialise or compete against each other across the different study sites.

## Discussion

Technology-based solutions have the potential to increase effectiveness of individualised quality healthcare without increasing healthcare delivery costs. Fall prediction and prevention is a research field where technology can be used to facilitate healthy ageing, well-being and independent-living [26]. iStoppFalls takes a novel approach to falls prevention and risk assessment for community-dwelling older people. This paper reports on the design of the innovative home-based iStoppFalls system for continuous monitoring and prevention of fall risk in older people. As part of the iStoppFalls consortium, falls researchers have worked with ICT experts and videogame developers to design a home-based fall risk assessment tool and exercise program for older people using innovative technologies. Existing research evidence was translated and implemented into sustainable technology-based self-management strategies using inexpensive mainstream entertainment equipment.

The technology-based iStoppFalls FRA enables objective assessments of balance, stepping, reaching and STS transfers in older people’s homes at regular time intervals [15, 16]. It was modelled on evidence-based screening tools such as QuickScreen and the SPPB [10]. It facilitates identification of fall risk factors for each individual, which could allow for individual tailoring of intervention strategies. Furthermore, the convenience of conducting the assessment in someone’s own home facilitates self-management and allows for small changes in fall risk to be detected sooner compared to generally more infrequent laboratory assessments. Further validation of this tool is needed using prospective designs to establish whether it can also predict future falls. In order to improve the ability of the tool to observe change in each user across the measure of fall risk factors, future studies could also explore using continuous data from the physical assessments.

The unique design and delivery of the iStoppFalls exercise program through technology makes it possible to offer both traditional strength training and innovative balance exergames, with additional cognitive challenges. The use of technology also allows new realms of falls prevention exercise programs to be explored through the gamification of exercise. Elements of entertainment can be employed to immerse people in exergames to reach new levels of exercise volumes (duration, frequency and intensity), motivation and long-term adherence. Furthermore, the possibility to provide immediate performance feedback enables the user to evaluate their current abilities and their progression over time, which is an essential feature to increase long-term adherence [8]. The feedback obtained from the SMM on distance walked could also act as a cue to take up a more active lifestyle. In addition to providing immediate performance feedback, iStoppFalls also explored the option for peers and potentially clinicians to interact with each other to open a line of communication whereby people may obtain assistance when needed. This has great potential to lessen the burden on the health care system and also to offer falls prevention services in remote communities without regular access to healthcare facilities.

## Conclusions

iStoppFalls provides a new concept for unobtrusive monitoring of fall risk and physical activity by using technologies in order to motivate and engage users. The concepts and methodologies integrated in the iStoppFalls system provide a valuable foundation for further research in these fields. Future research and development should focus on tailoring of the intervention program, incorporating regression as well as progression when people improve due to training or decline due to illness. Future research should also explore mechanisms to enhance long-term adherence by fully exploiting the use of technology (e.g. by combining information from exercise sessions and SMM-based daily life activity and mobility profiles). A multi-site randomised control trial has been completed in 160 healthy community-dwelling older people to establish feasibility and acceptability and determine the program’s efficacy on fall risk factors. The protocol for this study is registered with the Australian New Zealand Clinical Trials (ACTRN12614000096651) and the International Standard Randomised Controlled Trials (ISRCTN15932647) [28]. It is expected that the outcomes of iStoppFalls will be highly relevant to individuals, policy makers and healthcare industries.
